# Investigating the effect of dynamic traffic distribution on network-wide traffic emissions: An empirical study in Ningbo, China

**DOI:** 10.1371/journal.pone.0305481

**Published:** 2024-07-12

**Authors:** Shuichao Zhang, Jianan Shi, Yizhe Huang, Hao Shen, Kangkang He, Hongjie Chen

**Affiliations:** 1 Department of Traffic Engineering, Ningbo University of Technology, Ningbo, China; 2 Zhejiang Engineering Research Center of Digital Road Construction Technology, Ningbo, China; 3 Department of Traffic Engineering, Chang’an University, Xi’an, China; Southwest Jiaotong University, CHINA

## Abstract

Urban road traffic is one of the primary sources of carbon emissions. Previous studies have demonstrated the close relationship between traffic flow characteristics and carbon emissions (CO2). However, the impact of dynamic traffic distribution on carbon emissions is rarely empirically studied on the network level. To fill this gap, this study proposes a dynamic network carbon emissions estimation method. The network-level traffic emissions are estimated by combining macroscopic emission models and recent advances in dynamic network traffic flow modeling, namely, Macroscopic Fundamental Diagram. The impact of traffic distribution and the penetration of battery electric vehicles on total network emissions are further investigated using the Monte Carlo method. The results indicate the substantial effect of network traffic distribution on carbon emissions. Using the urban expressway network in Ningbo as an example, in the scenario of 100% internal combustion engine vehicles, increasing the standard deviation of link-level traffic density from 0 to 15 veh/km-ln can result in an 8.9% network capacity drop and a 15.5% reduction in network carbon emissions. This effect can be moderated as the penetration rate of battery electric vehicles increases. Based on the empirical and simulating evidence, different expressway pollution management strategies can be implemented, such as petrol vehicle restrictions, ramp metering, congestion pricing, and perimeter control strategies.

## 1. Introduction

The transportation industry is one of the major sources of carbon emissions, of which urban road transportation is the primary component. It is reported that urban road transportation contributes up to 95.3% of total carbon emissions of road transportation in New York [1], 77.6% in London [2], 86.5% in Tokyo [3] and 86.76% in China [4]. During driving and idling, internal combustion engine vehicles emit substantial amounts of carbon monoxide (CO), hydrocarbons (HC), nitrogen oxides (NOx), carbon dioxide (CO_2_), and particulate matter (PM). These emissions, particularly carbon dioxide and other greenhouse gases, contribute to global warming, increase climate system instability, and lead to more frequent extreme weather [5–7]. The key to managing and reducing vehicle emissions lies in accurately measuring vehicle emissions under various traffic conditions.

Emission models are usually categorized into macroscopic emission models and microscopic emission models. The macroscopic emission models estimate the level of transportation emissions in a larger area based on parameters such as vehicle characteristics, road network data and emission factors, mainly including COPERT [8–11], MOBILE [12], IVE [13], and EMFAC [14]. Microscopic emission models estimate emission levels at the micro-level of individual vehicles or traffic based on factors such as vehicle type, speed, acceleration, etc. Compared with macroscopic emission models, the microscopic emission models are more accurate and flexible, but the data collection process is difficult and time-consuming. Common microscopic emission models include CMEM [15], MOVES [16, 17], VT–Micro [18], HBEFA [19], and PHEM [20]. Currently, the COPERT, MOVES, and IVE are the most widely used emission models. Among these models, the COPERT model stands out for its simplified calculation process, reducing the need for complex modeling, and making it easier to use. Additionally, China’s emission standards align with the European system, making the emission factors calculated by the COPERT model more applicable to Chinese scenarios.

Network traffic emissions are influenced by various factors, including traffic patterns, road conditions, and congestion levels. A single emission model, such as COPERT or MOVES, faces challenges when directly estimating network emissions. Estimating network emissions requires a more comprehensive approach that considers these dynamic factors, which may go beyond the capabilities of a single emission model. Recently, the Macroscopic Fundamental Diagram (MFD)-based method has been validated for network-wide traffic carbon emission estimation [21–25]. The concept of MFD was initially proposed by Daganzo (2007) [26], and its existence and basic properties were verified through the measured data in Yokohama, Japan in 2008 [27]. The MFD-based methods exhibit the relationship between network traffic flow, density and speed [28], which is helpful in understanding the network traffic dynamics and improving the network traffic system. Some research estimated network-wide emissions by integrating the emission model with MFD. For example, Shabihkhani and Ghamami (2014) [21] integrated microscopic emissions models with MFD to estimate the aggregated network-wide emissions of greenhouse gases from vehicles. Saedi et al. (2020) [22] proposed a network-level emission modeling framework based on the network-wide fundamental diagram. Csikós et al. (2015) [23] used MFD to simulate traffic conditions and proposed a road network emission control, method to minimize network emissions. Ji et al. (2023) [24] proposed a carbon emission macroscopic fundamental diagram model (CE-MFD) to assess Shanghai expressway network-wide road emissions. Batista et al. (2022) [25] utilized aggregated traffic models based on the Macroscopic Fundamental Diagram to estimate network-wide emissions.

The urban road network is typically a heterogeneous system with an uneven distribution of traffic characteristics over time and space. However, to the best of our knowledge, the impact of network traffic heterogeneity on overall road network emissions is rarely considered when estimating the network-wide carbon emissions. To fill the gap, this paper aims to extend the existing emission MFD method and empirically investigates the effect of network heterogeneity on network carbon emissions from the perspective of traffic density heterogeneity. Specifically, the three sub-objectives are:

- To empirically estimate the network-level traffic carbon emissions by combining MFD with macroscopic emission models.
- To analyze and illustrate the effect of network traffic heterogeneity on network carbon emissions.
- To predict the network emissions under different levels of network heterogeneity and various penetration rates of electric vehicles.

The remainder of this paper is organized as follows: Section 2 introduces the research area and the data used in this study. Section 3 combines the carbon emission model with MFD to construct the carbon emission-MFD. Section 4 focuses on the effect of network traffic heterogeneity on network carbon emissions. Finally, discussion and conclusions are provided in Section 5.

## 2. Research area and data

Ningbo, located in northeast Zhejiang province, is one of the 15 sub-provincial cities in China with nearly 10 million residents. The urban expressway network in Ningbo is selected as the research area, which includes BeiHuan, NanHuan, JiChang, and DongHuan expressways. The key characteristics of these selected urban expressway are presented in Table 1. Each expressway has two to five lanes (one direction), with a speed limit of 80 km/h. JiChang expressway has a length of 43.5 km and is equipped with 75 fixed traffic checkpoints. BeiHuan and NanHuan expressways have lengths of 21.3 km and 21.8 km, with 84 and 117 checkpoints respectively. DongHuan expressway has a length of 16.1 km and is equipped with 28 checkpoints.

**Table 1:** Basic properties of selected urban expressway.

Expressway	Length [km]	Number of Lanes	Number of checkpoints	Number of Ramps	Speed Limit [km/h]
**BeiHuan**	21.3	2–5	84	16	80
**NanHuan**	21.8	3–5	117	12	80
**JiChang**	43.5	3–5	75	20	80
**DongHuan**	16.1	3–5	28	12	80

**Fig 1:**
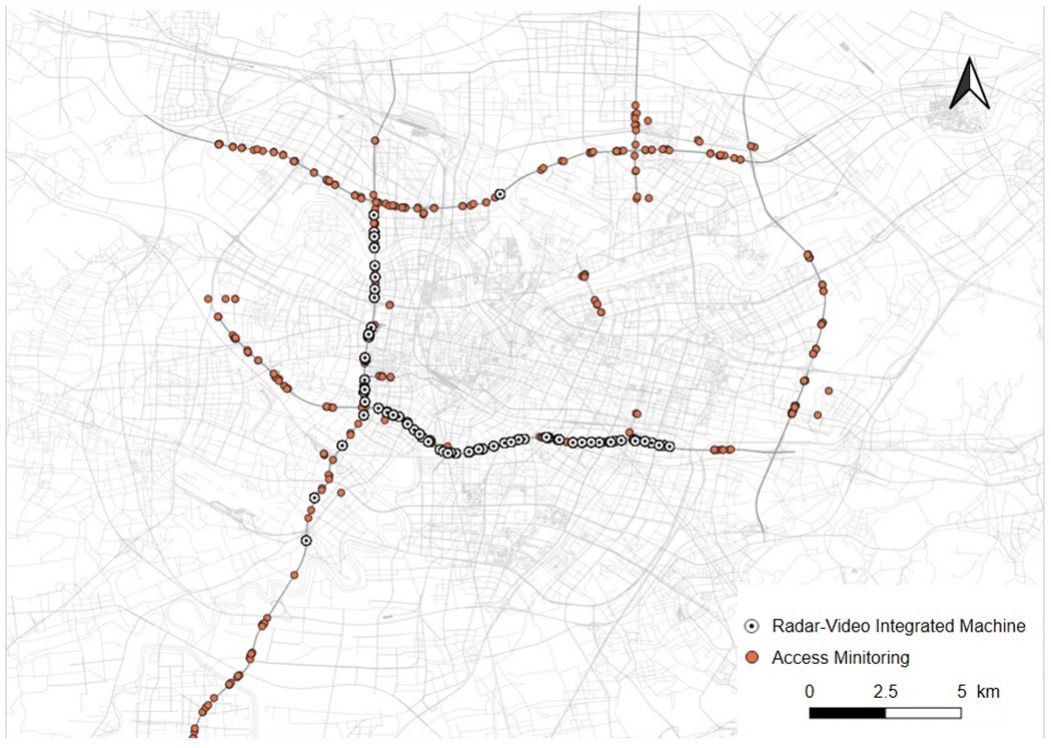
Representation of Ningbo urban expressway network and the position of each traffic checkpoint. (Base map and data from OpenStreetMap and OpenStreetMap Foundation).

Fig 1 shows the map of research area and the location of each traffic checkpoint. The traffic checkpoints are installed at approximately 600-meter intervals along the selected urban expressways. In this study, two types of traffic data collected by multiple devices of traffic checkpoints, namely radar-video integrated machine data and access monitoring data, are used to empirically analyze the dynamic traffic conditions and carbon emissions. The radar-video integrated machines are mainly distributed along the main line of expressway. The dataset contains device ID, longitude and latitude, flow, speed, and time. Access monitoring devices are installed on the main line of urban expressways and on/off ramps. The dataset contains attributes including device ID, longitude and latitude, flow, and time. The empirical data were collected for one week (June 20-June 26, 2022) from Ningbo Traffic Authorities.

## 3. Macroscopic modelling of network traffic and network emission

### 3.1 MFD model

Macroscopic fundamental diagram (MFD) is an inherent attribute of road network [28]. The shape of MFD exhibits the recurrent relationship between network traffic flow and network traffic density, reflecting network-wide traffic flow dynamics. The empirical estimation of MFD requires few data input and has low computation cost, which has now been widely used in various aspects of traffic management and control, such as route guidance [29], perimeter control [30], congestion pricing [31], and parking control [32]. Based on the generalized traffic definition proposed by Edie (1963) [33], the network flow *Q*(t), density *K*(t) and speed *V*(t) can be calculated as Eqs (1)–(3), respectively.

$$Q(t)=\frac{\sum_{i=1}^{I}q_{i}(t)\times l_{i}}{\sum_{i}^{I}l_{i}}$$ (1)

$$V\left(t\right)=\frac{\sum_{i=1}^{I}v_{i}(t)\times l_{i}}{\sum_{i}^{I}l_{i}}$$ (2)

$$K\left(t\right)=\frac{\sum_{i=1}^{I}k_{i}(t)\times l_{i}}{\sum_{i}^{I}l_{i}}$$ (3)

where, *I* is the total number of links within the network; *q*_*i*_*(t)* is the flow of link *i* at time *t*; *l*_*i*_ is the length of link *i*; *v*_*i*_*(t)* is the speed of link *i* at time *t* and *k*_*i*_*(t)* is the density of link *i* at time *t*.

### 3.2 Carbon emission model

Since July 1, 2020, China has fully implemented the National VI emission standard (National VI). The National VI emission standard follows the framework structure of the European emission standard, and previous studies have proven the effectiveness of the COPERT model in this context [34]. Therefore, this paper will utilize the COPERT model to calculate the carbon emission of the Ningbo expressway network.

Traffic emission pollutants originate from three phases of vehicle operation: thermal stabilized engine operation (hot), warming-up phase (cold start) and fuel evaporation. Among these phases, hot emissions are the main phase of pollutant generation and emission, and this phase includes vehicle operation and idling Previous studies have shown that the emissions from cold start and evaporative are much smaller compared to hot emission [35]. Thus, this study mainly focuses on the hot emission stage.

The COPERT model assumes that emission factors corresponding to hot engine operation depend only on average speed. Based on the average speed of vehicles, the generic power train models can be used to analyze the energy consumption of Internal Combustion Engine Vehicle (ICEV) and Battery Electric Vehicles (BEV), which have been investigated by [36]. According to the results of [36], the relationship between average speed and energy consumption exhibits a convex function. The low-carbon speed is about 30–40 km/h for a BEV with generation and the speed range is about 50–60 km/h for an ICEV.

The fuel consumption of an ICEV and the electricity consumption of a BEV are denoted as EF_ICEV_ (g/km-ln) and EF_BEV_ (Wh/km-ln), which can be extracted from [36] as follows:

$$\begin{array}{ll} EF_{ICEV} & =5.862\times 10^{-13}\times V^{8}-3.051\times 10^{-10}\times V^{7}+8.71\times 10^{-8}\times \\ & V^{6}-1.17\times 10^{-5}\times V^{5}+0.0009218\times \\ & V^{4}-0.04334\times V^{3}+1.195\times V^{2}-18.44\times V+171.2 \end{array}$$ (4)

$$\begin{array}{ll} EF_{BEV} & =1.134\times 10^{-12}\times V^{8}-6.972\times 10^{-10}\times V^{7}+1.786\times 10^{-7}\times \\ & V^{6}-2.472\times 10^{-5}\times V^{5}+0.002008\\ & \times V^{4}-0.09751\times V^{3}+2.783\times V^{2}-43.56\times V+424.1 \end{array}$$ (5)

where, *V* is the average speed of the vehicles within the road segment.

Due to the truck restriction policy, few diesel vehicles can be found on the urban expressway in Ningbo. This study focuses on the carbon emissions of gasoline vehicles, where emission factor of CO_2_ can be directly proportional to gasoline consumption. The CO_2_ emissions of gasoline vehicles can be obtained by multiplying the total amount of consumed gasoline by the CO_2_ conversion coefficient $\text{C}F_{CO_{2}}$ [37], which is calculated as follows:

$$\begin{array}{ll} CF_{CO_{2}} & =\text{Average}\;\text{low}\;\text{heat}\;\text{value}\times \text{Carbon}\;\text{content}\;\text{per}\;\text{unit}\;\text{of}\;\text{calorific}\;\text{value}\times \\ & \text{Fraction}\;\text{of}\;\text{Carbon}\;\text{Oxidized}\times 44/12 \end{array}$$ (6)

The relevant factors [38] are given in Table 2.

**Table 2:** Conversion coefficient of gasoline.

Fuel	Average Low heat value [KJ/kg,m^3^]	Carbon content per unit of calorific value [t-C/TJ]	Fraction of Carbon Oxidized
**Gasoline**	43070	18.90	0.98

Consequently,

$$CF_{CO_{2}}=43070\times 18.90\times 0.98\times 10^{-6}\times 44/12=2.9251$$ (7)

Combining the conversion factor of gasoline $\text{C}F_{CO_{2}}$ with Eq (4), the CO2 emissions per kilometer per lane of one ICEV, $\text{E}F_{ICEV,CO_{2}}$ (kg/km-ln), can be calculated as:

$$\begin{array}{l} EF_{ICEV,CO_{2}}=CF_{CO_{2}}\times 10^{-3}\times EF_{ICEV}\\ =2.9251\times 10^{-3}\times (5.862\times 10^{-13}\times V^{8}-3.051\times 10^{-10}\times \\ V^{7}+8.71\times 10^{-8}\times V^{6}-1.17\times 10^{-5}\times V^{5}\\ +0.0009218\times V^{4}-0.04334\times V^{3}+1.195\times V^{2}-18.44\times V+171.2) \end{array}$$ (8)

The amount of CO2 emitted by electricity consumption is calculated by multiplying the emission factor by the electricity consumption [39]. At present, the Chinese national power grid emission factor is 0.5810 kg/kWh [40]. Thus, the CO_2_ emissions per kilometer per lane of one BEV (kg/km-ln) can be described as:

$$\begin{array}{ll} EF_{BEV,CO_{2}} & =0.581\times 10^{-3}\times (1.134\times 10^{-12}\times V^{8}-6.972\times 10^{-10}\\ & \times V^{7}+1.786\times 10^{-7}\times V^{6}-2.472\times 10^{-5}\times V^{5}+0.002008\times \\ & V^{4}-0.09751\times V^{3}+2.783\times V^{2}-43.56\times V+424.1) \end{array}$$ (9)

### 3.3 Carbon emission macroscopic fundamental diagram (CE-MFD)

In this section, to understand the relationship between network carbon emissions and network dynamic traffic flow characteristics, the carbon emission macroscopic fundamental diagram (CE-MFD) is constructed by combining the macroscopic fundamental diagram of expressway and macroscopic traffic carbon emission model.

Denote p as the penetration rate of vehicles on the road, p_*ICEV*_ and p_*BEV*_ represent the proportion of ICEVs and BEVs, respectively. Then we have:

$$p_{ICEV}=1-p_{BEV}$$ (10)

$$q_{i,ICEV}(t)=q_{i}(t)\times p_{ICEV}$$ (11)

$$q_{i,EV}(t)=q_{i}(t)\times p_{BEV}$$ (12)

Following Ji et al., (2023), the average carbon emission of ICEVs in the network in time period t per unit length per lane, E_ICEV_(t) and the average carbon emissions of BEVs in the network in time t per unit length per lane E_BEV_(t), are calculated as Eqs (13) and (14):

$$\;E_{ICEV}(t)\;=\sum_{i=1}^{I}EF_{ICEV,CO_{2},i}(t)\times q_{i,BEV}(t)\;\times l_{i}/\sum_{\text{i}=1}^{\text{I}}l_{i}$$ (13)

$$E_{BEV}(t)\;=\sum_{i=1}^{I}EF_{BEV,CO_{2},i}(t)\times q_{i,BEV}(t)\times l_{i}/\sum_{\text{i}=1}^{\text{I}}l_{i}$$ (14)

The average network emissions in the time t per unit length per lane E(t) can then be calculated by summing up the average carbon emissions of ICEVs and BEVs as follows:

$$E(t)=E_{ICEV}(t)+E_{BEV}(t)$$ (15)

For a given road network with a total length of L, the total network emissions during the time period T can be estimated as follows:

$$E_{total}\;=\Sigma_{t\in T}E(t)L$$ (16)

## 4. Effect of dynamic traffic distribution on network emissions

### 4.1 The dynamic traffic distribution within the network

The urban road network is typically a heterogeneous system with an uneven distribution of traffic characteristics over time and space. Previous studies have indicated that unevenly distributed traffic demand is the main cause of network traffic heterogeneity, which can significantly affect the network traffic capacity [41]. The traffic distribution within the urban expressway network is investigated based on the radar-video integrated machine data collected from checkpoints shown in Fig 1. For different average network traffic density (i.e., 10, 15 and 20 veh/km-ln), the link density of each expressway segment between adjacent ramps is divided into 15 groups in ascending order. The distribution of link density can indicate the level of network traffic heterogeneity under different values of average network traffic density.

**Fig 2:**
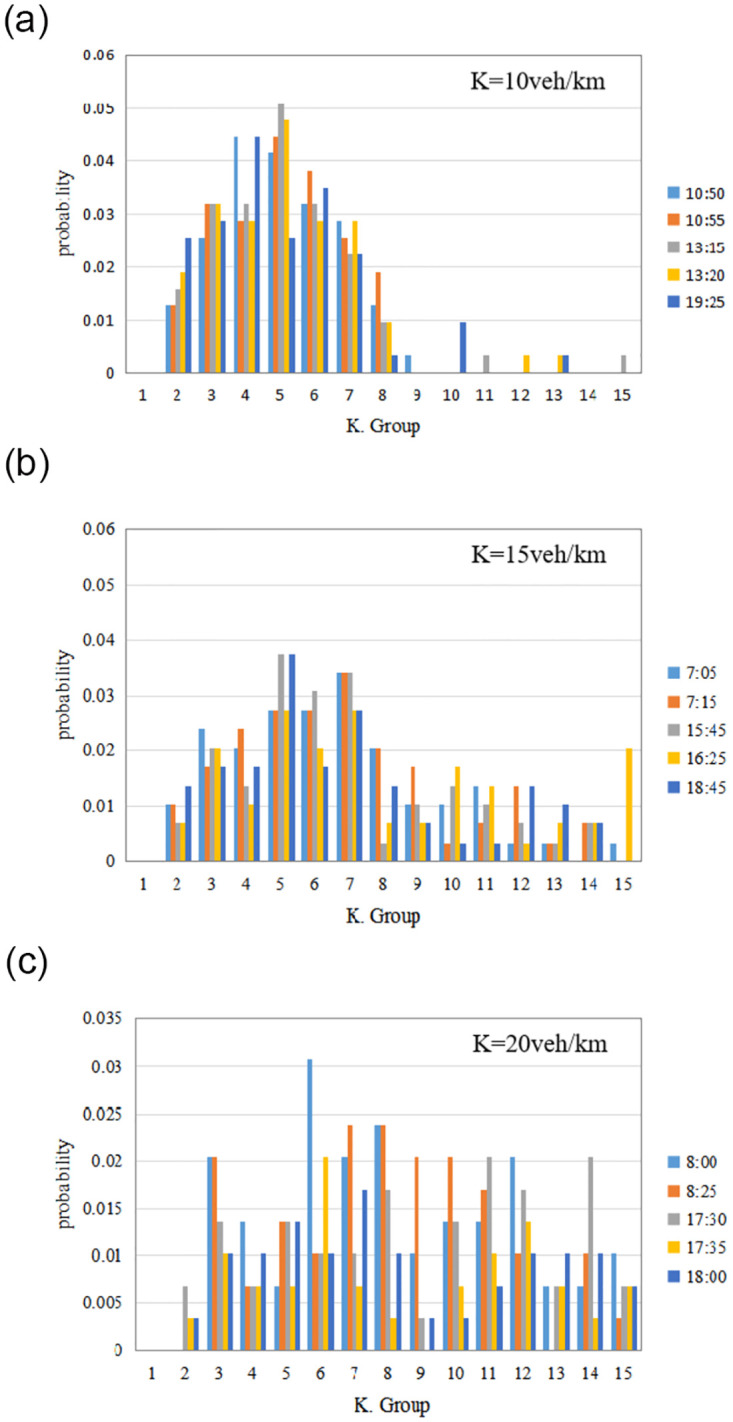
Histograms for different values of network traffic density in June 20, 2022. (a) K = 10veh/km-ln. (b) K = 15veh/km-ln. (c) K = 20veh/km-ln.

Fig 2 shows the network traffic distribution of Ningbo urban expressway on workdays (June 20, 2022). Similar to the results in [42], for each average network traffic density group, the distribution of link density is uneven within the whole expressway network. As more parts of the network become congested, the distribution of link density is shifted to the right.

To quantify the dynamic network traffic heterogeneity for a certain time period t, the standard deviation of link density within the network σ(t) [43] is used:

$$\sigma \left(\text{t}\right)=\sqrt{\frac{1}{\text{I}}\sum_{i=1}^{I}{\left(k_{i}(t)-\frac{\sum_{i=1}^{I}k_{i}(t)}{I}\right)}^{2}}$$ (17)

The value of σ(t) can reflect the degree of traffic distribution imbalance within the network. Based on Eqs (1)–(3) and (17), MFDs (density-flow diagrams) for different directions of expressways in Ningbo are estimated based on 5-min time intervals.

**Fig 3:**
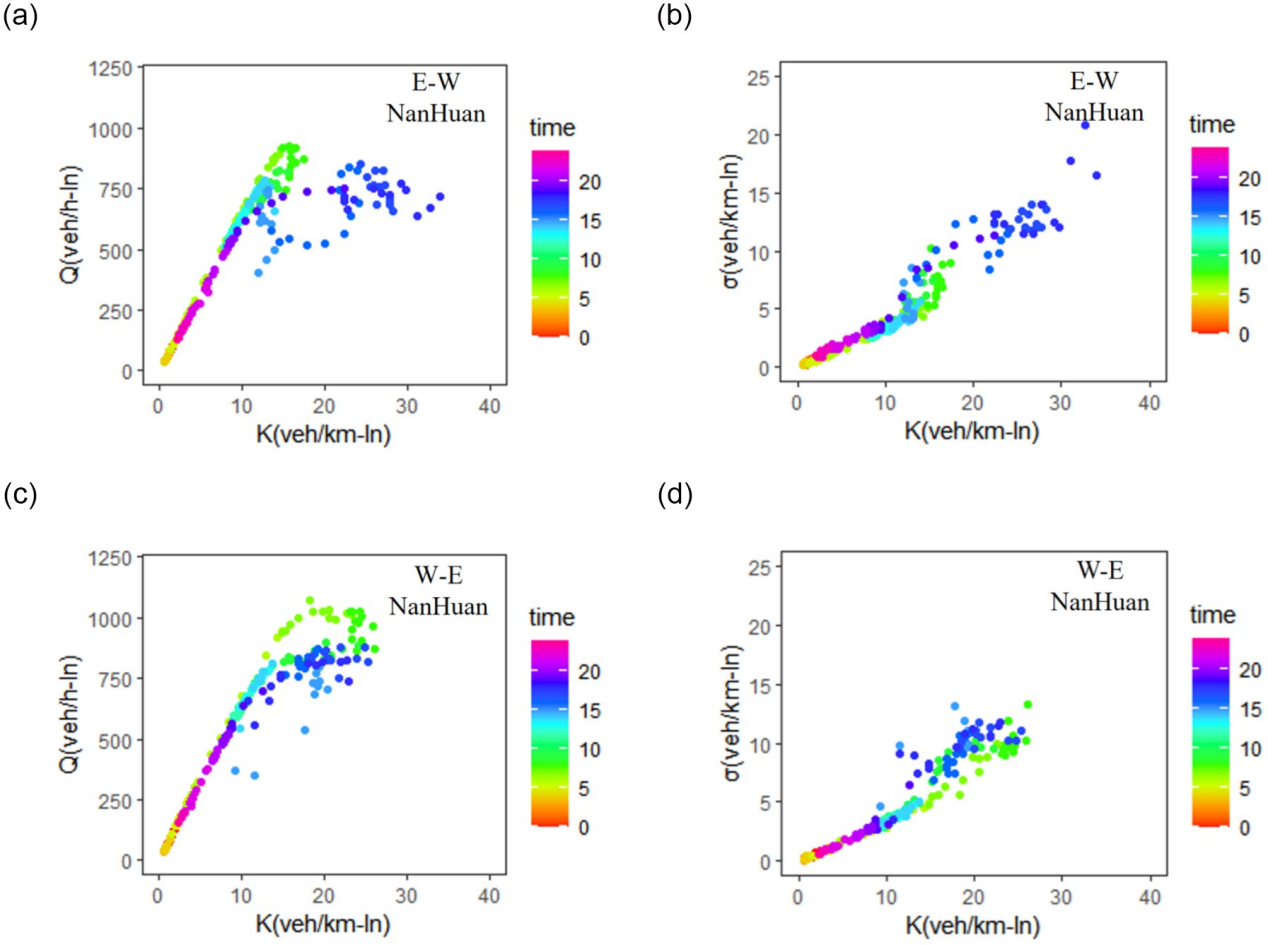
The relationship between network flow and density (left) and the relationship between network density heterogeneity and density (right) for different directions of expressways. (a),(b) the east-west direction of NanHuan. (c),(d) the west-east direction of NanHuan.

Fig 3 presents the dynamic relationship between network traffic flow Q and density K, as well as the dynamic relationship between network density heterogeneity σ and network traffic density K in NanHuan expressways. As shown in the Figure, the MFDs exhibit distinct hysteresis loops, indicating the unevenly distributed traffic within the network during both traffic loading and uploading process. For a given network accumulation of vehicle, the average network flow Q substantially decreases with the increase of network traffic heterogeneity σ(t). According to the time-varying values of σ(t), the East-West direction of NanHuan expressway has higher traffic heterogeneity, especially during the evening peak hours. The values of network traffic heterogeneity σ(t) range from 0 to 15 veh/km-ln in this case.

The east-west direction of NanHuan Expressway has higher density heterogeneity during the evening peak hours than the morning peak hours, resulting in 38.7% capacity drop for the same network traffic density. For the west-east direction of NanHuan Expressway, the traffic heterogeneity can account for 10% capacity drop during the evening peak hours.

### 4.2 Dynamic analysis of network carbon emissions

#### 4.2.1 Estimated results of network carbon emissions

**Fig 4:**
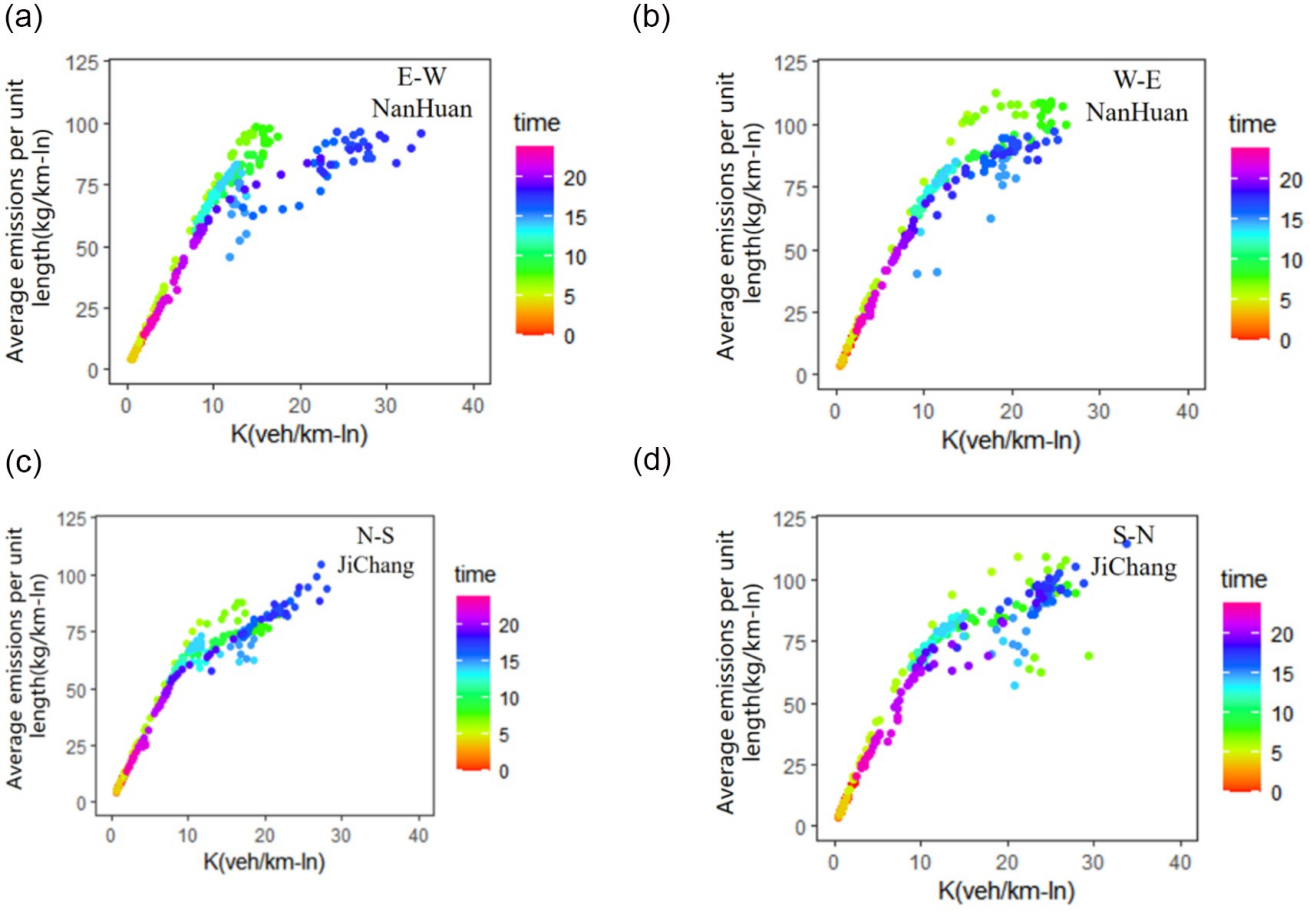
CE-MFD in different directions of expressways. (a) the east-west direction of NanHuan. (b) the west-east direction of NanHuan. (c) the north-south direction of JiChang. (d) the south-north direction of JiChang.

Based on the CE-MFD method proposed in Section 3.3, the time-varying emissions of different directions of each expressway can be estimated using Eqs (10)–(17). As presented in Fig 4(a)–4(d), during the morning peak, traffic emissions in the west-east direction of NanHuan are substantially higher than those in the east-west direction. Meanwhile, traffic emissions in the south-north direction of JiChang are substantially higher than those in the north-south direction. The hysteresis loops exist when modeling the relationship between network traffic density and emissions, indicating the substantial effect of network traffic heterogeneity on the total network emissions.

The traffic emissions characteristics of different expressway directions exhibit substantial difference during the morning and evening peak hours. In the east-west direction of NanHuan Expressway, the maximum traffic emission is 98.23 kg/km-ln during the morning peak hours, which is reached when the average traffic density is 14.78 veh/km-ln. The maximum traffic emission during the evening peak hours is 96.62 kg/km-ln, which is reached when the average traffic density is 24.07 veh/km-ln. In the west-east direction of the NanHuan Expressway, the morning peak emission is 112.58 kg/km-ln and the evening peak emissions is 97.4 kg/km-ln. The heterogeneity of traffic emissions in different directions is also observed on the JiChang Expressway. In the north-south direction, the morning traffic emissions peak is 88.26 kg/km-ln and the value increase to 104.89 kg/km-ln during the evening peak. In the south-north direction of JiChang Expressway, the highest traffic emissions during the morning peak is 109.09 kg/km-ln and the maximum emission is 114.1 kg/km-ln during the evening peak.

#### 4.2.2 The impact of average network traffic heterogeneity

To further investigate the effect of heterogeneity on network emissions, different scenarios of network traffic heterogeneity are simulated based on the Monte Carlo method. By substituting the average density of each link $\sum_{\text{i}=1}^{\text{I}}{\text{k}}_{\text{i}}\text{(t)}/I$ with average network density *K*(*t*) in Eq (17), the relationship between network traffic heterogeneity and average network density can be further transformed into the following form:

$$K{\left(t\right)}^{2}+{\bar{\sigma }}^{2}=\frac{1}{I}\sum_{i=1}^{I}k_{i}{\left(t\right)}^{2}$$ (18)

Unlike the time-varying indicator of network heterogeneity σ(t), the value of $\bar{\sigma }$ represents the average heterogeneity of the road network and remains constant throughout the day in this section. For a given value of $\bar{\sigma }$, such as 0, 5, 10, and 15 veh/km-ln, different groups of link density can be simulated based on the Monte Carlo method by assuming the distribution of road segment density. In this study, the distribution of link density is assumed to follow a normal distribution with a mean of *K*(*t*) and a standard deviation of $\bar{\sigma }$. The simulation samples with deviations between $K{\left(t\right)}^{2}+\bar{\sigma }{\left(t\right)}^{2}$ and $\frac{1}{I}\Sigma_{\text{i}=1}^{\text{I}}k_{i}{\left(t\right)}^{2}$ less than 5% and each link density *k*_*i*_(*t*) > 0 were selected.

**Fig 5:**
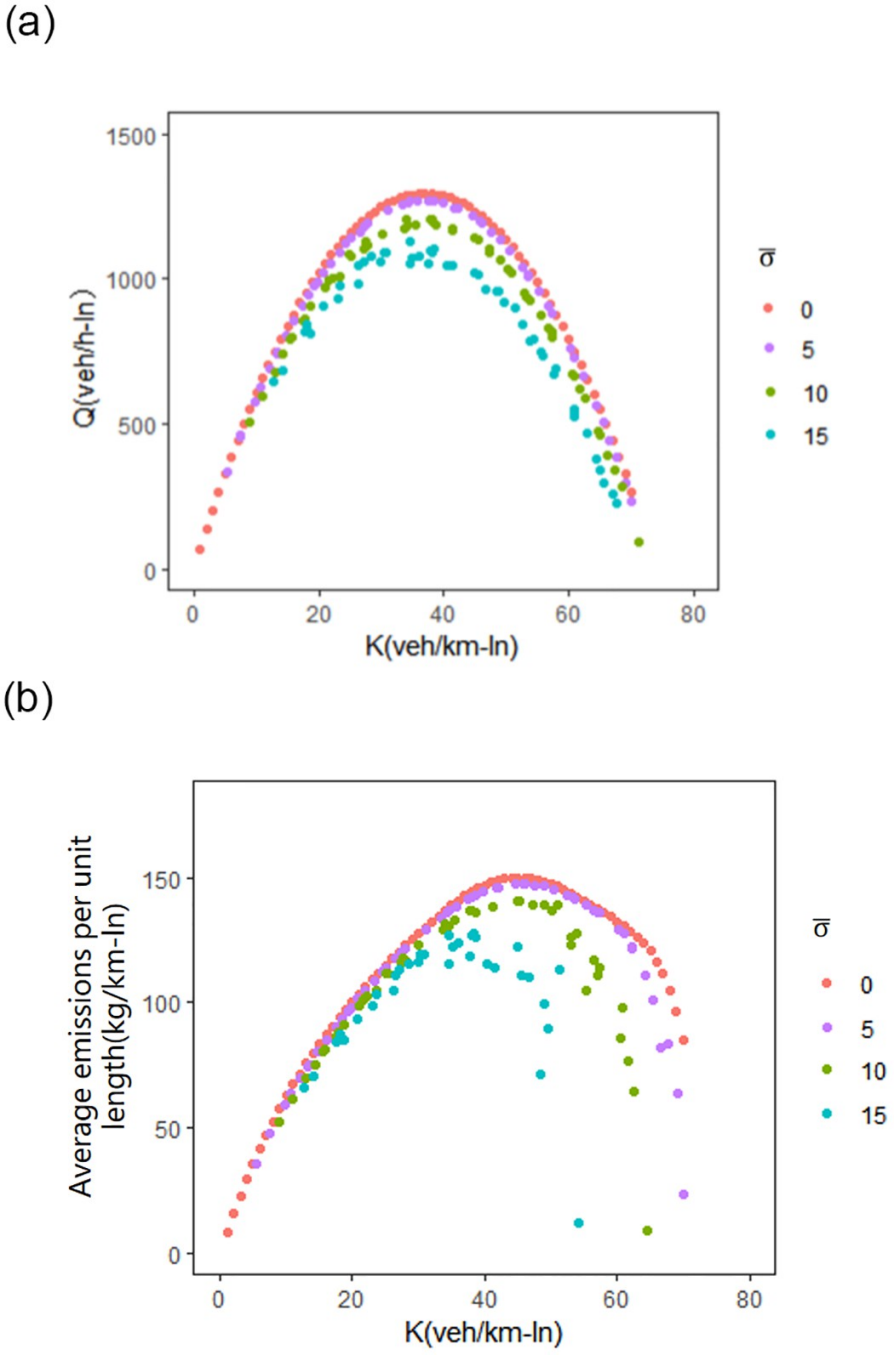
The data are distinguished according to the standard deviation. (a) relationship between the average network flow and the average network traffic density. (b) network emissions and the average network traffic density.

The impact of average network traffic heterogeneity on MFD and CE-MFD is investigated by assuming different average standard deviation of network traffic density $\bar{\sigma }$ (0, 5, 10 and 15 veh/km-ln). The results of MFD and CE-MFD under different levels of network traffic heterogeneity are shown in Fig 5. It can be observed that higher values of network heterogeneity can lead to lower values of both network flows and network emissions for the same average network traffic density.

When the network density reaches the critical density (37 veh/km-ln), the maximum network flow Q is close to 1,300 veh/h-ln for a small average network traffic heterogeneity ($\bar{\sigma }=5$ veh/km-ln), while Q is close to 1,130 veh/h-ln as the network traffic heterogeneity increase to 15 veh/km-ln. The maximum value of network carbon emissions per unit length is reached when the average network traffic density is 46 veh/km-ln. When the average network traffic heterogeneity is 5 veh/km-ln, maximum carbon emission is approximately 150 kg/km-lane. The maximum emissions can be reduced to 130 kg/km-lane as the average network heterogeneity increases to 15 veh/km-ln.

#### 4.2.3 The impact of the number of road segments and BEV penetration

**Fig 6:**
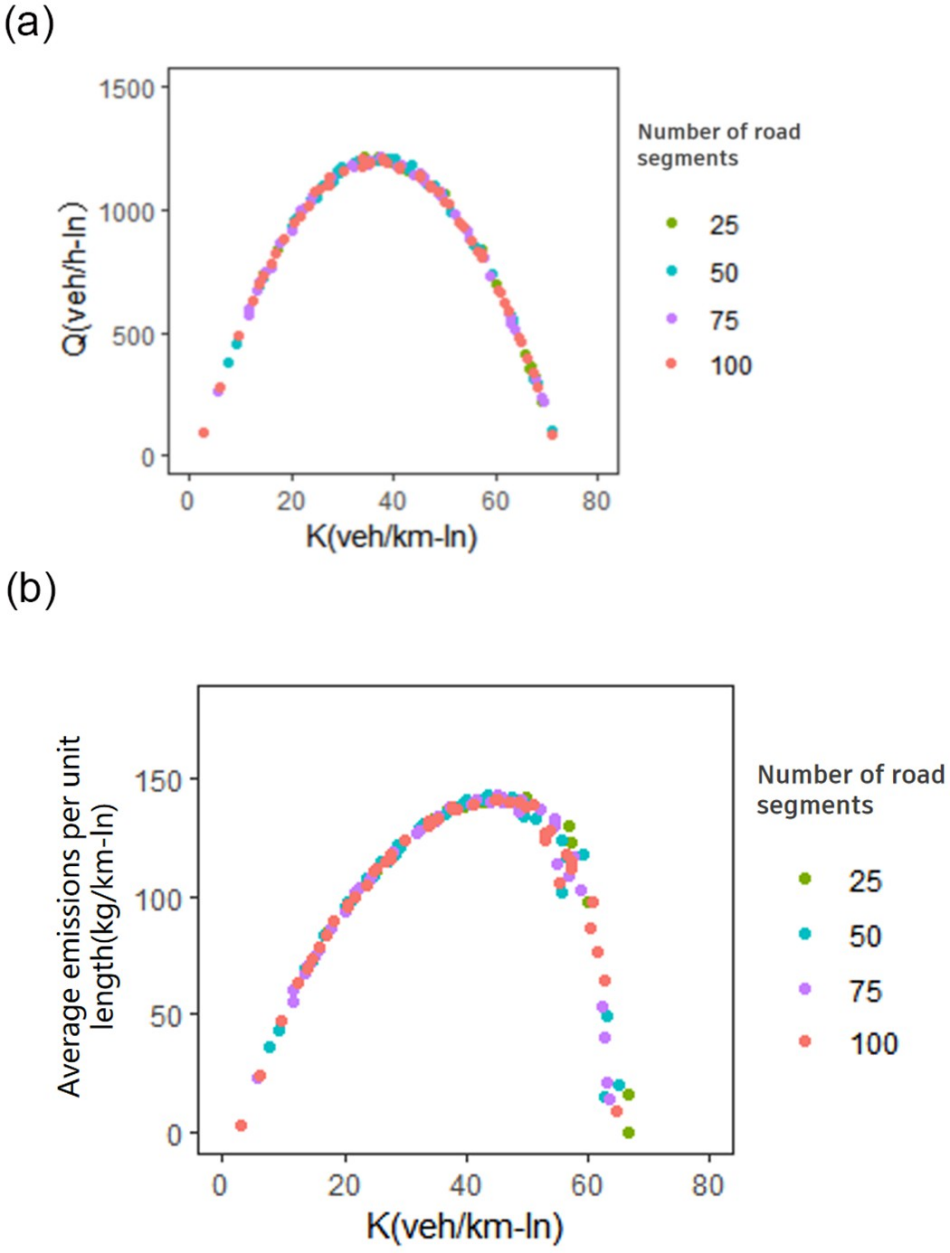
The data are distinguished according to the total number of links in the network I. (a) relationship between the average network flow and the average network traffic density. (b) network emissions and the average network traffic density.

To test the sensitivity results of estimated MFD and CE-MFD to the number of road segments within the network, a sensitivity analysis is performed based on the Monte Carlo method. The average network heterogeneity $\bar{\sigma }$ is assumed constant (10 veh/km-ln) and the shapes of MFD and CE-MFD are estimated for the given number of road segment (25, 50, 75 and 100 segments). As shown in Fig 6, when the number of road segments increases, the shapes of MFD and CE-MFD present no distinct changes. Therefore, the results of estimated network flow and network emissions are not sensitive to number of segments within the study network.

**Fig 7:**
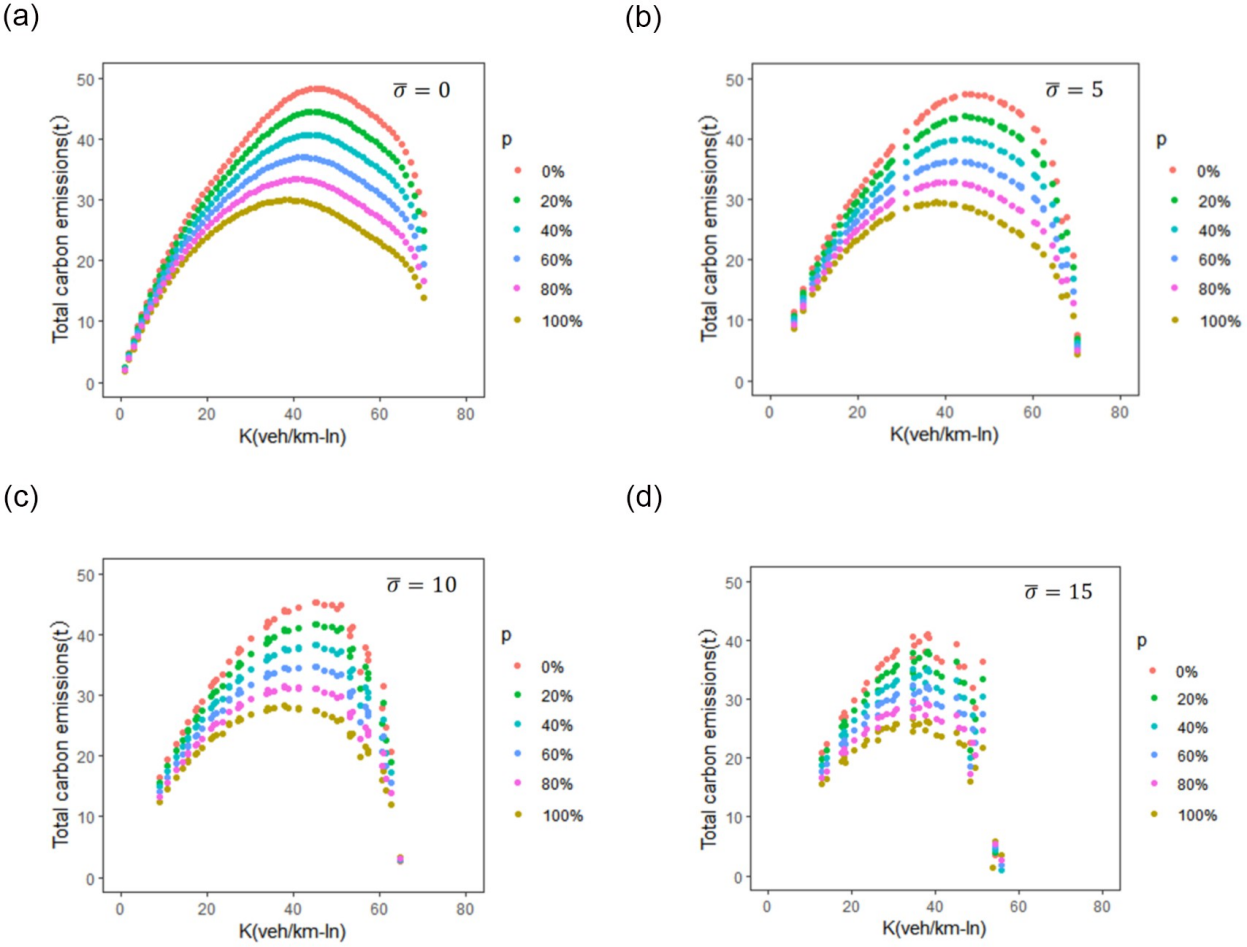
Impact of BEV penetration on emissions under different road network heterogeneity.

The impact of BEV penetration on emissions is further investigated under different road network heterogeneity. The total network carbon emissions of different BEV penetration rates *p*_*BEV*_ (0%, 20%, 40%, 60%, 80% and 100%) is calculated based on Eqs (15) and (16). Fig 7(a)–7(d) show the CE-MFD plots of different *p*_*BEV*_ with density standard of 0, 5, 10, and 15 veh/km-ln, respectively. The results of critical values for different groups are presented in Table 3.

**Table 3:** Traffic emissions for different network heterogeneity and BEV penetration rate.

Network traffic heterogeneity $\bar{\sigma }$ [veh/km-ln]	BEVs penetration rate *p*_*BEV*_	K_m_ [veh/km-ln]	Q_m_ [veh/h-ln]	The highest value of emissions [t]	Average emissions per vehicle [g/km]
0	0%	46.00	1,216.58	48.24	280.86
	20%	45.00	1,232.68	44.41	264.31
	40%	44.00	1,246.89	40.63	247.31
	60%	42.00	1,269.63	36.92	235.42
	80%	41.00	1,278.17	33.33	217.72
	100%	39.00	1,289.56	29.89	205.26
5	0%	46.12	1,191.61	47.44	275.48
	20%	44.52	1,217.11	43.67	262.71
	40%	44.52	1,217.11	39.95	240.33
	60%	42.14	1,242.38	36.25	230.38
	80%	39.87	1,259.74	32.72	219.79
	100%	37.99	1,271.53	29.47	207.75
10	0%	45.37	1,132.66	45.31	267.46
	20%	45.37	1,132.66	41.74	246.39
	40%	45.37	1,132.66	38.18	225.38
	60%	45.03	1,139.59	34.62	205.90
	80%	37.98	1,206.96	31.42	221.56
	100%	37.98	1,206.96	28.29	199.49
15	0%	36.95	1,107.42	40.78	295.58
	20%	36.95	1,107.42	37.90	274.70
	40%	36.95	1,107.42	35.02	253.83
	60%	36.95	1,107.42	32.14	232.95
	80%	36.95	1,107.42	29.26	212.08
	100%	36.95	1,107.42	26.38	191.21

Increasing the penetration rate of battery electric vehicles (BEVs) can substantially reduce carbon emissions from the network. According to the life-cycle estimation in this study, if the network traffic is evenly distributed ($\bar{\sigma }=0$), increasing the penetration rate of BEVs from 0% to 100% can reduce the maximum total network carbon emissions from 48.24 t to 29.89 t, resulting in a nearly 38% reduction in emissions.

The slope of the curve can well reflect the marginal effect of car accumulation on the total network emissions. As shown in Fig 7, when the traffic flow density is less than the critical density, the slope of the curve is positive and gradually decreasing, indicating that the accumulation of vehicles within the network will increase the network emissions. However, when the network traffic density exceeds the critical density, the slope of the curve becomes negative, which indicates that the network emissions decrease as the number of vehicles within the network increases.

As the network becomes increasingly unbalanced, the number of congested links within the network also increases, resulting in higher emissions per vehicle in those congested segments. However, the average emissions per single vehicle within the entire network may not necessarily increase. For example, when the penetration of BEVs is 20%, the average emission per vehicle is 264.31 g/km if the traffic is evenly distributed within the network ($\bar{\sigma }=0$ veh/km-ln). As the indicator of traffic heterogeneity $\bar{\sigma }$ increase to 5 veh/km-ln and 10 veh/km-ln, the average emission per vehicle decrease to 262.71 g/km and 246.39 g/km, respectively. The average emission per vehicle reaches 274.70 g/km when the $\bar{\sigma }$ is 15 veh/km-ln. Meanwhile, there is a rapid decline in total traffic flow within the network. As a result, although the traffic emissions increase in congested segments, the overall carbon emissions of the road network decrease.

In the scenario of 100% internal combustion engine vehicles (ICEV), increasing the standard deviation of link density from 0 to 15 veh/km-ln can result in nearly 8.9% network capacity drop (from 1,216.58 veh/h-ln to 1,107.42 veh/h-ln) and 15.5% total network carbon emissions reduction (from 48.24 t to 40.78 t). The impact of network traffic heterogeneity on the total network carbon emissions can be moderated as the penetration rate of battery electric vehicles increases.

## 5. Discussion and conclusions

This paper extends the existing carbon emission MFD method and investigates the relationship between network carbon emissions and dynamic traffic distribution using both empirical and simulated data. The empirical results of total network carbon emissions are consistent with [44] in terms of magnitude, which demonstrates the effectiveness of our estimation method. Compared with another empirical study of network carbon emissions estimation in the Shanghai expressway [24], we observe more distinct hysteresis loops when modeling the relationship between network traffic density and network emissions in the Ningbo expressway network. The effect of different levels of network traffic heterogeneity and the penetration of battery electric vehicles (BEVs) on network emissions is further analyzed based on the Monte Carlo method.

Our finding shows that as the network becomes increasingly unbalanced, the number of congested links within the network also increases, resulting in higher emissions per vehicle in those congested segments. Meanwhile, there is a rapid decline in total traffic flow within the network. As a result, although the emissions per vehicle increase, the overall carbon emissions of the road network decrease in the Ningbo case. Thus, using average network flow alone to estimate the network emissions in previous studies can result in substantial deviations in carbon emission estimates. The heterogeneity distribution of traffic demand within the road network should also be considered while estimating network traffic emissions.

Although the results of this study may be case-specific, the proposed analysis framework of estimating network carbon emissions based on network traffic heterogeneity can be effectively used for other megacities. Based on the empirical and simulating evidence in this research, different urban expressway management strategies at various stages of BEV policies can be implemented such as petrol vehicle restriction, ramp metering, congestion/emission pricing, and perimeter control strategies.
